# Co-expression of POU4F2/Brn-3b with p53 may be important for controlling expression of pro-apoptotic genes in cardiomyocytes following ischaemic/hypoxic insults

**DOI:** 10.1038/cddis.2014.452

**Published:** 2014-10-30

**Authors:** V Budhram-Mahadeo, R Fujita, S Bitsi, P Sicard, R Heads

**Affiliations:** 1 Medical Molecular Biology Unit, UCL Institute of Child Health, London, UK; 2 Cardiovascular Division, Faculty of Life Sciences and Medicine, Kings College London, London, UK

## Abstract

Cardiomyocyte death following ischaemic/hypoxic injury causes irreversible damage to cardiac function and contributes to chronic diseases such as heart failure. Understanding the mechanisms associated with myocyte loss under these conditions can help to identify strategies to minimise/abrogate such detrimental effects. The p53 protein can induce apoptosis or cell cycle arrest, but effects on cell fate depend on interactions with other regulators such as POU4F2/Brn-3b (Brn-3b), which co-operates with p53 to increase the expression of pro-apoptotic genes. In contrast, the related POU4F1/Brn-3a (Brn-3a) blocks p53-mediated apoptosis but co-operates with p53 to enhance cell cycle arrest. In this study, we showed that permanent coronary artery ligation in mouse hearts, which induced apoptotic markers, activated caspase-3 and -8 and necroptosis markers; RIP-1 and -3 also increased Brn-3b and Brn-3a expression. However, Brn-3a was only detected in uninjured myocardium but not at the site of injury, whereas Brn-3b showed generalised increase, including within the infarct zone. Conversely, p53 was detected in the infarct zone and in some cells adjacent to the site of injury but not in uninjured myocardium. Co-localisation studies showed Brn-3a co-expression with p53 in cardiomyocytes adjacent to the infarct zone, whereas Brn-3b was co-localised with p53 in the infarct zone only. Increased Brn-3b and p53 correlated with elevated expression of pro-apoptotic target genes, Bax, Noxa and PUMA, whereas cleaved caspase-3 confirmed the presence of apoptotic cells within this region of the injured heart. Similarly, simulated ischaemia/reoxygenation (sI/R) injury in neonatal rat ventricular cardiomyocytes (NRVM) and heart derived H9c2 myoblasts increased Brn-3b, p53 as well as apoptotic genes, and this was associated with enhanced apoptosis. Furthermore, targeted reduction of Brn-3b using shRNA caused reduction in pro-apoptotic Bax and Noxa proteins, even though p53 expression remained intact, suggesting that Brn-3b is important for controlling the fate of the myocardium in the injured heart.

During myocardial infarction (MI), prolonged ischaemia and/or hypoxia can cause the death of cardiomyocytes that adversely affect cardiac function.^1, 2^ Modes of cell death implicated in such cell loss include necrosis, programmed necrosis (necroptosis), apoptosis and autophagy^2, 3^ and these vary in relation to the site of injury. For example, necrotic cells are seen primarily within the central ischaemic zone, whereas apoptotic cardiomyocytes are detected in the infarct zone and ‘border zone' myocardium, adjacent to the site of injury. Interestingly, apoptotic cells are detectable within 2 h after injury, induced by coronary artery ligation^4, 5, 6, 7^ but are also implicated in longer-term cardiomyocyte loss that contributes to heart failure.^7^

However, although necrotic cell death is associated with changes such as loss of inner mitochondrial membrane potential, MPTP opening and inflammation, programmed cell death such as necroptosis and apoptosis require dynamic changes in gene expression. Thus, necroptosis is accompanied by activation of receptor-interacting proteins, RIP1/3^8, 9^ whereas apoptosis is characterised by increased expression of pro-apoptotic proteins and activation of caspases.^10, 11, 12^ Therefore, DNA-binding transcription factors (TFs) that activate or repress the transcription of genes that encode such proteins will be important for regulating these processes.^13^ However, the effects of TFs are modulated by interaction with other proteins in the transcriptional complex.^13, 14^

The Brn-3b and Brn-3a are related, but distinct proteins, encoded by different genes, that belong to the POU (Pit-Oct-Unc) family of TFs.^15^ These proteins exist as two isoforms that differ in size because of the presence of an additional N′ terminal domain in the longer proteins, for example, Brn-3b(l), that is not found in the shorter Brn-3b(s),^16, 17^ but the functions of these distinct isoforms are still to be elucidated. Brn-3b share high homology (>90%) with Brn-3a in the DNA-binding POU domain,^18^ which means that these TFs bind to similar DNA sites in target gene promoters. However, limited sequence similarity outside of the POU domain^15^ causes distinct effects on gene expression and cell fate, which is also affected by the interaction with other cellular regulators. For example, Brn-3b drives growth and proliferation in some cells by enhancing the expression of cell cycle proteins such as cyclin D1 and CDK4.^19, 20^ Accordingly, elevated Brn-3b in solid tumours, such as childhood neuroblastomas and breast cancers, correlates with increased cyclin D1 levels.^17, 21, 22, 23^ However, if growth-promoting Brn-3b is co-expressed with p53, which inhibits cell growth, such conflicting signals drive apoptosis and under such conditions, Brn-3b interacts and co-operates with p53 to increase transcription of pro-apoptotic Bax, thereby increasing cell death.^24^

In contrast, the related Brn-3a supports survival and differentiation in neuronal cells by transactivating genes encoding anti-apoptotic proteins (e.g., Bcl-2 and Bcl-X_L_) and neuronal proteins (e.g., neurofilament; *α*-internexin; SNAP25).^25, 26, 27^ However, Brn-3a also interacts with p53 but blocks the transactivation of pro-apoptotic genes whilst co-operating with p53 to enhance the expression of the cell cycle inhibitor, p21^cip1/waf1^.^28, 29, 30^ As Brn-3b and Brn-3a have antagonistic effects on survival when co-expressed with p53, these proteins will be particularly important under conditions that induce/activate p53. p53 is considered a tumour suppressor protein because of its inhibitory effects on cell growth. It is found at low levels in most cells under normal conditions but can be rapidly induced and activated in response to injury/cellular stresses including ischaemia, hypoxia, DNA damage and oxidative stress.^31, 32, 33^ When activated, p53 can give rise to diverse cellular effects including cell cycle arrest, DNA repair or apoptosis, depending on which of its target genes are activated. For example, under mild stress, p53 increases the expression of p21^cip1/waf1^, associated with senescence and differentiation, whereas extensive damage or severe stress stimulates pro-apoptotic p53 target genes, for example, Bax, Noxa and PUMA, thereby triggering apoptosis.^33, 34^ The diverse effects of p53 on cell fate are highly regulated by its interaction with other cellular proteins, such as Brn-3a, which blocks p53-mediated apoptosis but co-operates with p53 to promote senescence/differentiation,^29, 30^ whereas Brn-3b co-operates with p53 to enhance apoptosis.^24^

Brn-3b and Brn-3a expression have previously been reported in cardiomyocytes,^35^ whereas p53 is undetectable in healthy adult hearts but has been implicated in different cardiac pathologies, including hypoxia and ischaemia/reperfusion (I/R) injury.^36, 37^ Despite conflicting reports regarding its role(s) in the heart, p53 is now considered to be important for mediating the responses in injured cardiomyocytes^36, 37, 38, 39^ because p53 knockout (KO) mice show increased survival following left coronary artery occlusion.^37^ Similarly, cardiac-specific reduction of p53 attenuates apoptosis in the infarct border zone after coronary artery ligation and reduces ventricular remodelling during the late phase of MI.^39^ Furthermore, p53 is induced in response to hypoxia in isolated cardiomyocytes,^36^ whereas pro-apoptotic p53 target genes are implicated in the death of injured cardiomyocytes.^40, 41, 42, 43, 44, 45^

In this study, we demonstrate that Brn-3b and Brn-3a are increased in the injured heart and co-localise with p53 in distinct regions in relation to the site of injury. Furthermore, Brn-3b co-expression with p53 within the infarct zone correlated with cleaved caspase-3 positivity and increased pro-apoptotic genes, PUMA, Noxa and Bax. *In vitro* studies show that simulated ischaemia/reoxygenation (sI/R) also induces Brn-3b and p53 in primary cultures of neonatal rat ventricular cardiomyocytes (NRVM) or rat embryonic-heart-derived H9c2 cells and this correlates with increased pro-apoptotic genes and cleaved caspase-3. Finally, short-hairpin RNA (shRNA)-mediated reduction of Brn-3b protein is sufficient to attenuate the expression of pro-apoptotic genes and increase cell survival, even though p53 expression is unchanged. The implications of these findings in relation to determining cell fate in the injured heart are discussed.

## Results

### Brn-3b and Brn-3a TFs are induced in cardiomyocytes following coronary artery ligation

**Figure 1:**
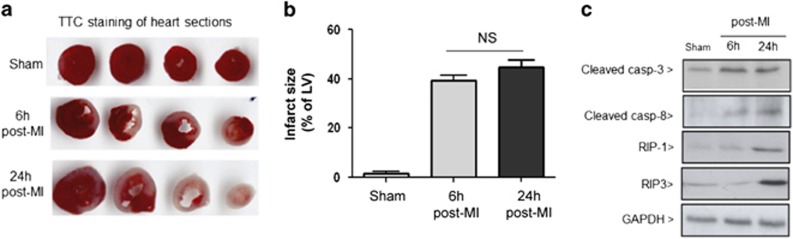
(**a**) Triphenyltetrazolium chloride staining showing infarct size (white zone) in sections taken from hearts after sham surgery or at sham, 6 h and 24 h post permanent LAD ligation surgery. (**b**) Myocardial infarct size was expressed as a percentage of the left ventricle area (mean±S.E.M. (*n*=4/groups)). (**c**) Representative immunoblots showing the expression of apoptotic markers, cleaved casapse-8 and caspase-3 and necroptosis markers, RIP1 and RIP3 in sham hearts compared with tissues taken from hearts at 6 h and 24 h following permanent LAD ligation surgery (*n*=3 per group)

MI was induced in mouse hearts by permanent ligation of the left anterior descending (LAD) coronary artery and changes in tissue viability were assessed by triphenyltetrazolium chloride staining (Figure 1a). Quantification of non-viable tissue showed substantial increase (>40%) in cell death by 6 and 24 h after ligation (Figure 1b). To examine the cell death pathways that were activated in the injured heart, we analysed for changes in apoptotic markers (cleaved caspase-3 or -8) and necroptosis markers (RIP-1/-3).^46, 47^ Figure 1c shows that cleaved caspase-3 and -8 were increased by 6 h post ligation and remained elevated at 24 h, thereby confirming that apoptosis occurred during early time points following permanent coronary artery ligation.^4, 48^ RIP-3 and RIP-1 were only detected at 24 h post MI, suggesting that necroptosis occurs at later time points.

**Figure 2:**
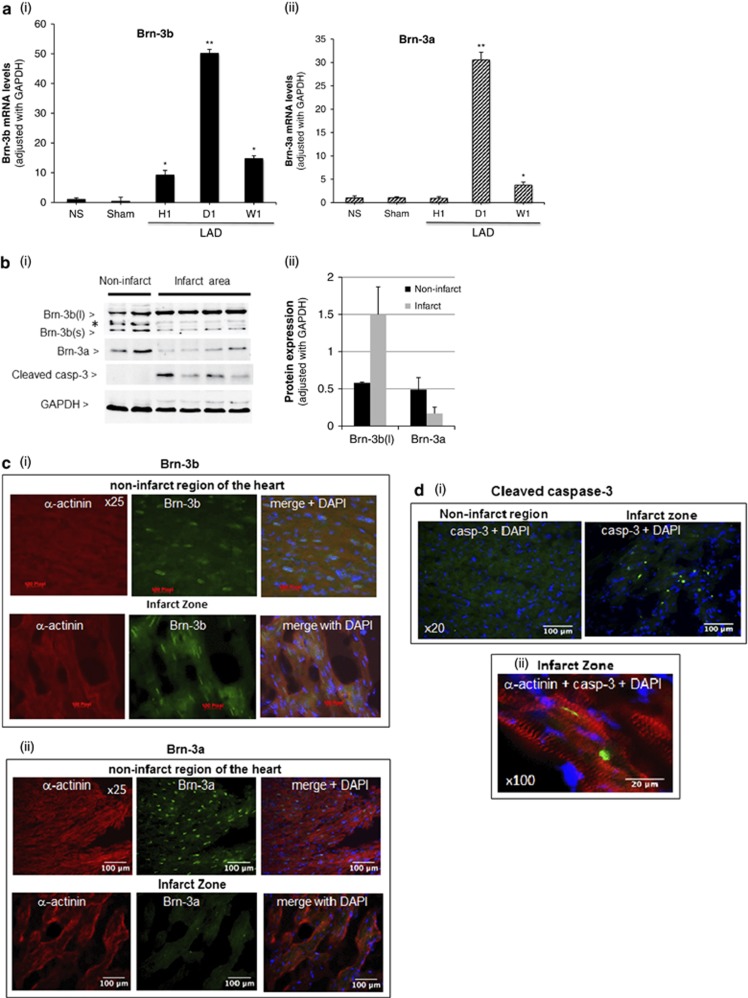
(**a**) qRT-PCR data showing changes in mRNA encoding Brn-3b (i) and Brn-3a (ii) in hearts following ligation of the LAD after 1 h (H 1), 1 day (D 1) and 1 week (W1), compared with control hearts taken from mice with either no surgical intervention (NS) or sham surgery (sham). Relative mRNA levels were normalised to the housekeeping gene, GAPDH, (to adjust for variations between different samples) and fold changes, calculated using ΔΔCT method, were expressed relative to sham control (set at 1). Values shown represent the mean±S.D. of >5 independent hearts at each time point. Statistical significance was assessed by Student's *t*-test. *P<0.05, **P<0.01, ***P<0.001. (**b**) (i) Representative immunoblots showing Brn-3b and Brn-3a proteins in tissues taken from non-infarct area and infarct zone of hearts following coronary artery ligation. Each lane represents proteins expressed in a different heart. The longer Brn-3b isoform is shown as Brn-3b(l) and Brn-3b(s) represent the shorter protein. * shows the position of an additional band that is slightly larger than Brn-3b(s) and is regulated in a similar manner. Cleaved caspase-3 was used to determine apoptotic changes in these regions of the heart, whereas GAPDH levels were used to show variability in protein levels. (ii) Bar graph showing quantification of Brn-3b and Brn-3a protein expression in non-infarct tissue compared with the infarct zone, using densitometry of immunoblots, which were normalised to GAPDH levels for each sample (mean±S.E.M. of >5 independent samples). (**c**) Representative immunofluorescence images of transverse sections of mouse hearts harvested at 1 day after ligation showing Brn-3b or Brn-3a (green), co-stained with sarcomeric *α*-actinin (red), as a marker of cardiomyocytes. Non-infarcted images refer to tissues in uninjured region of the heart, whereas the infarct zone represents tissues around the site of injury. Merged images show the overlay of Brn-3b or Brn-3a with *α*-actinin as well as DAPI staining, which identify the nuclei of cardiomyocytes in these sections. Images were captured at × 25 magnification. (**d**) (i) Immunofluorescent images showing cleaved caspase-3 protein expression in the infarct zone but not in the non-infarcted tissue in hearts at 1 day after coronary artery ligation. DAPI staining indicates the location of cell nuclei within this section. (ii) Images ( × 100) showing co-staining of activated casp-3 (green) with *α*-actinin (red) in cardiomyocytes within the infarct zone of hearts, 1 day after LAD ligation. DAPI staining (blue) indicates the cell nuclei in the sections

As Brn-3b and Brn-3a TFs are expressed in cardiomyocytes and have antagonistic effects on cell survival, we next used quantitative reverse transcriptase polymerase chain reaction (qRT-PCR) to analyse for changes in mRNA encoding these TFs in whole hearts following coronary artery ligation, compared with sham controls. Figure 2a(i) shows that increased Brn-3b mRNA was observed as early as 1 h after coronary artery ligation; maximally expressed after 1 day and reduced by 1 week although levels remained higher than baseline. In contrast, Brn-3a mRNA was unchanged at 1 h, increased significantly after 1 day but decreased after 1week.

Cardiomyocyte fate is affected by its location in relation to the infarct zone; hence, we next analysed protein levels in tissues isolated from the infarct zone and from uninjured regions of the heart. Figure 2b(i) shows representative Brn-3a and Brn-3b expression in non-infarcted and infarcted heart tissue (Figure 2b(ii)). Brn-3a protein was only detected in non-infarct regions but not in the infarct zone (Figure 2b), whereas Brn-3b showed more complex changes. Thus, uninjured (non-infarct) tissue expressed Brn-3b(l) and Brn-3b(s) isoforms as well as an intermediate band that was regulated in a similar manner to Brn-3b(s). In contrast, the infarct zone expressed increased Brn-3b(l) but reduced Brn-3b(s). Although cleaved caspase-3 was not detected in the non-infarct myocardium, significant increases in the infarct area confirmed the presence of apoptotic cells and suggest that Brn-3b(l) may be specifically increased in injured myocardium.

To determine cellular localisation in cardiomyocytes, co-immunostaining was carried out for each protein and *α*-actinin, on transverse sections of 1 day post-ligation hearts. Representative immunofluorescent images demonstrated that both TFs co-localised with *α*-actinin (Figures 2c(i) and (ii)). Brn-3a was primarily expressed in the non-ischaemic regions of the heart but not in the infarct zone, whereas Brn-3b was detected in the non-ischaemic regions of the heart (Figure 2c(i)) (top panel) and in the infarct zone (bottom panel). Increased cleaved caspase-3 was detected within the infarct zone but not in the non-ischaemic areas (Figure 2d(i)), whereas co-staining with *α*-actinin confirmed that cardiomyocytes in the infarct zone were undergoing apoptosis (Figure 2d(ii)).

### Co-expression of p53 with Brn-3b or Brn-3a in distinct regions of injured hearts

**Figure 3:**
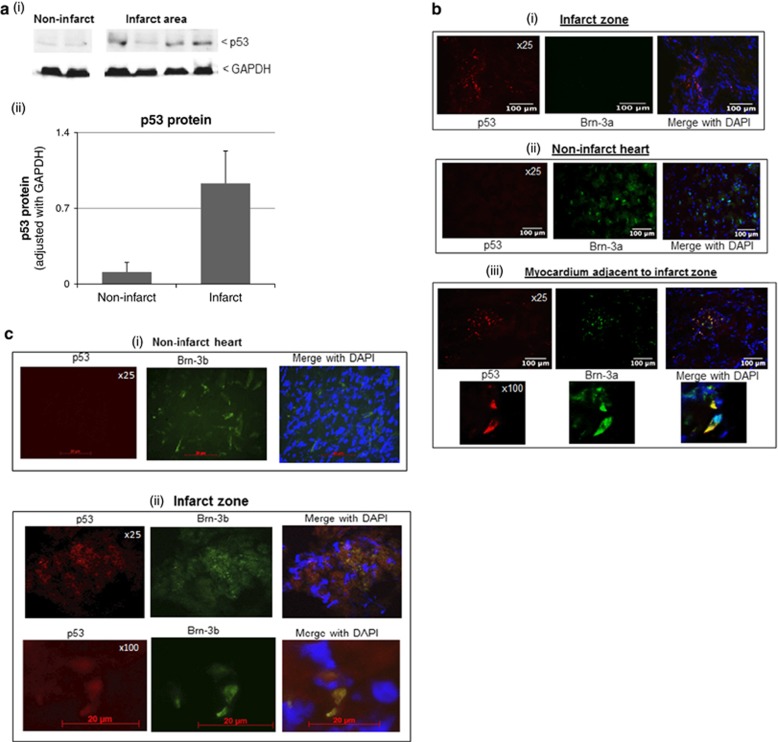
(**a**) Representative immunoblots showing p53 protein levels in tissues taken from uninjured (non-infarct) tissue or infarct zone, whereas GAPDH expression was consistent across samples. (ii) Graph showing changes in p53 protein in the infarct zone, compared with non-infarct tissue. Proteins have been normalised to GAPDH levels and represent mean±S.E.M. of >5 samples. (**b** and **c**) Representative immunofluorescence images of heart sections showing co-staining for p53 (red) and either Brn-3a (green) (**b**) or Brn-3b (**c**) in the infarct zone (i) or non-infarcted regions (ii), whereas co-staining in myocytes adjacent to the infarct zone was only shown for Brn-3a and p53 (iii). Merged images show the overlay of either Brn-3a or Brn-3b with p53, whereas DAPI identifies the nuclei of cardiomyocytes in these sections

As p53 controls genes that affect cell survival in injured tissue, we next analysed p53 protein levels in the infarcted or uninjured heart tissues. Figure 3a shows that p53 protein was undetectable in the uninjured myocardium but was expressed in the infarct zone. Since Brn-3a and Brn-3b cause differential effects on cell survival when co-expressed with p53, co-immunostaining was carried out on heart sections at 1 day after coronary artery ligation. Figure 3b shows that p53 protein was expressed around the infarct zone but was not detected in the non-ischaemic regions of the heart, so did not overlap with Brn-3a apart from in a subset of cells adjacent to the infarct zone, which co-expressed both Brn-3a and p53 (Figure 3b(iii)). In contrast, Brn-3b co-localized with p53 in cells within the infarct zone (Figure 3c(ii)) but was also expressed in the non-ischaemic regions of the heart, which lacked p53 (Figure 3c(i)). However, this antibody did not distinguish between the different Brn-3b isoforms.

**Figure 4:**
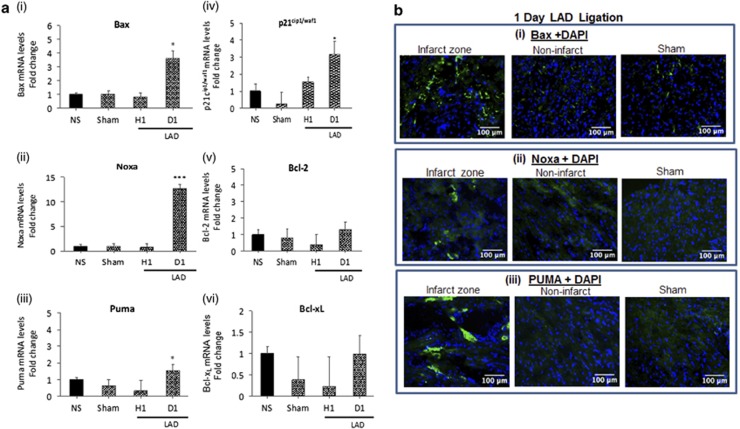
(**a**) qRT-PCR data showing changes in mRNA that encode different target genes including Bax (i); Noxa (ii); Puma (iii); p21^cip1/waf1^ (iv); Bcl-2 (v) and (vi) Bcl-xL in injured mouse heart following coronary artery ligation for 1 h or 1 day, compared with NS or sham control hearts. mRNA levels were normalised to GAPDH mRNA and fold changes expressed relative to the NS control heart (set at 1). Values shown represent mean±S.D. from >5 independent hearts at each time point with statistical significance assessed using Student's *t*-test (*P<0.05, **P<0.01, ***P<0.001). (**b**) Immunofluorescence images of heart sections (1 day after coronary artery ligation) stained with antibodies to recognise (i) Bax (ii) Noxa or (iii) PUMA proteins. Representative images of staining in the infarct zone or non-infarct zone in ligated hearts compared with sham control hearts. DAPI staining was used to show location of nuclei in the sections

To test how changes in p53 and Brn-3a or Brn-3b affected gene expression linked to survival, in injured heart, qRT-PCR was used to analyse for changes in mRNA encoding target genes regulated by these factors. Figure 4a shows increased expression of pro-apoptotic Bax and Noxa and to a lesser extent PUMA mRNA in hearts at 1 day after LAD artery ligation, when compared with NS or sham control hearts, whereas anti-apoptotic, Bcl-2 and Bcl-X_L_ mRNA remained unchanged. p21^cip1/waf1^ mRNA was also increased in these hearts. Fluorescent images of immunostained heart sections showed strong signals for Bax and Noxa proteins in the infarct zone of the heart, with weaker staining in the non-infarcted myocardium, particularly for Noxa (Figure 4b). Although there were small increases in PUMA mRNA, this protein was readily detected by immunostaining in the infarct zone but was undetectable in the non-ischaemic regions or in sham control heart. Thus, increased Brn-3b and p53 in the infarcted myocardium correlated with elevated expression of pro-apoptotic genes.

### Simulated ischaemia/hypoxia+reoxygenation increases Brn-3b, p53 in NRVM cultures

**Figure 5:**
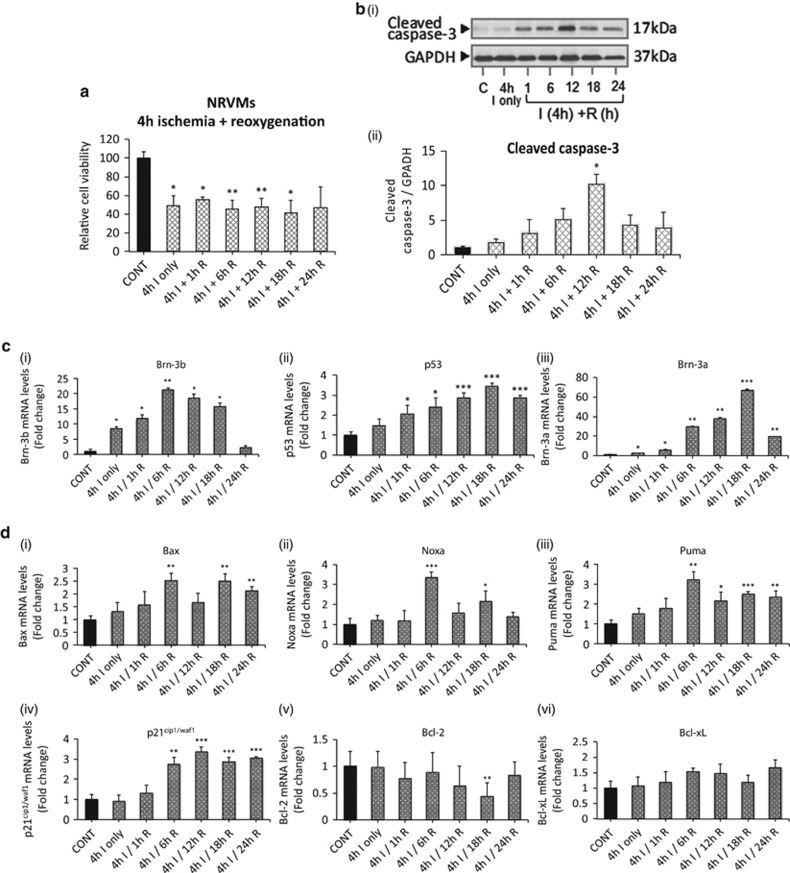

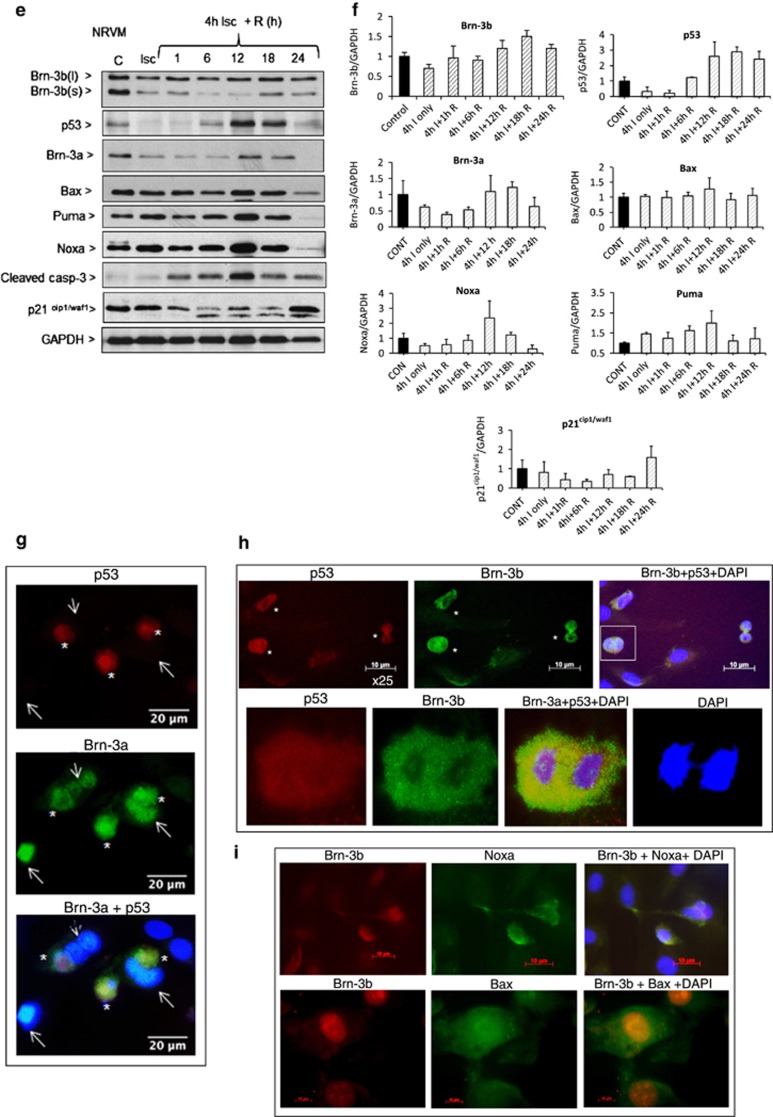
(**a**) MTT assay showing changes in percentage of viable cells in NRVM cultures subjected to simulated ischaemia/hypoxia (I) followed by reoxygenation for different times. Values are expressed as percentage decrease of viable cells compared with untreated control cells (set at 100%). Statistical significance was obtained using Student's *t*-test with *P<0.05, **P<0.01. (**b**) (i) Representative immunoblot showing cleaved caspase-3 protein levels in NRVM cultures subjected to 4 h ischaemia/hypoxia alone or with reoxygenation at specified times. (ii) Bar graph showing changes in cleaved caspase-3 protein expression in different samples following densitometry of immunoblots, normalised to GAPDH levels for each sample (mean±S.E.M. of >5 independent samples). (**c**) qRT-PCR data showing changes in mRNA encoding Brn-3b (i), p53 (ii) and Brn-3a TFs, in NRVM cultures, treated as indicated. Gene expression was normalised to GAPDH mRNA and expressed as fold changes compared with untreated control (set at 1). Values represent mean±S.E.M. of>3 independent experiments at each time point and Student's *t*-test shows significance, *P<0.05, **P<0.01, ***P<0.001. (**d**) qRT-PCR analysis showing changes in mRNA encoding Bax (i), Noxa (ii), Puma (iii), p21^cip1/waf1^ (iv), Bcl-2 (v) and (vi) Bcl-xL in NRVM cultures following ischaemia/hypoxia −/+ reoxygenation for different periods. mRNA levels were normalised to GAPDH mRNA and fold changes calculated using ΔΔCT method. Values are expressed as fold change relative to the untreated control cells (set at 1). Values represent mean±S.D. from >3 independent hearts at each time point with statistical significance assessed using Student's *t*-test (*P<0.05, **P<0.01, ***P<0.001). (**e**) Representative immunoblots showing changes in protein expression in NRVM cultures under different conditions. C=untreated control; Isc=4 h ischaemia/hypoxia; Isc+R=4 h ischaemia followed by reoxygenation for the time indicated. (**f**) Quantification of protein expression by densitometry and normalisation to GAPDH levels. Data are expressed as mean±S.E.M. fold change vs untreated cells of >3 independent experiments. (**g**) Representative immunofluorescent images ( × 25) showing expression of p53 (top panel-red) or Brn-3a (middle panel-green) in NRVMs treated with 4 h ischaemia+12 h reoxygenation. Merged images (lower panel) show co-localisation of p53 and Brn-3a, whereas DAPI identified the cell nuclei. * indicates specific cells that co-express Brn-3a and p53, whereas arrows show the cells that express Brn-3b but not p53. (**h**) Co-localisation of p53 (red) or Brn-3b (green) in NRVMs following ischaemia (4 h) and reoxygenation (12 h) at × 25 magnification. Merged images (right panel) show co-localisation of p53, Brn-3b and DAPI with * indicating cells that co-express Brn-3b and p53. Lower panels show higher magnification ( × 100) of a representative cell that co-expresses Brn-3b and p53 and show evidence of nuclear division (seen by DAPI stain). (**i**) Representative images showing Brn-3b (red) co-localised in cells that express either Noxa (green-top panels) or Bax (bottom panels) after ischaemia/reoxygenation

Next, *in vitro* studies were carried out in NRVM that express endogenous Brn-3a and Brn-3b proteins, whereas p53 can be induced by simulated ischaemia/reoxygenation (sI/R). Methylthiazolyldiphenyl-tetrazolium bromide (MTT) assays demonstrated reduced cell viability in NRVM cultures following 4 h simulated ischaemia/hypoxia (sI), which was sustained after reoxygenation (Figure 5a). However, immunoblotting showed that the apoptotic marker, cleaved caspase-3, was undetectable following sI but was expressed after reoxygenation (Figure 5b), suggesting that apoptotic cell death occurs after reoxygenation, whereas the loss of viable cells after sI may be associated with necrotic death.

qRT-PCR showed increased Brn-3b mRNA in NRVM cultures following sl which continued to rise (≥20-fold), 6–18 h after re-oxygenation (sI/R) (Figure 5c(i)) whereas p53 and Brn-3a mRNA were unchanged after sI but increased after 6 h re-oxygenation (Figure 5c(ii+iii)). Immunoblots demonstrated that both Brn-3b(l) and Brn-3b(s) protein isoforms were expressed in control cells (Figures 5e and f) but only the longer Brn-3b(l) was increased following sI or sI/R whereas Brn-3b(s) was reduced. On the other hand, p53 protein was only increased following 6 h sI/R whereas Brn-3a protein was reduced during sI and at immediately after sI/R but was increased at 12–18 h.

Interestingly, mRNA encoding pro-apoptotic Bax, Noxa and PUMA were increased following sI/R, whereas pro-survival genes, Bcl2 and Bcl-X_L_, remained unchanged (Figure 5d). To confirm changes in protein expression, immunoblots were carried out. Figure 5e shows increased Noxa and PUMA proteins following reoxygenation but Bax protein did not change significantly even though mRNA was higher (Figures 5d–f).

In view of the opposite effects of Brn-3a and Brn-3b on cell survival when co-expressed with p53, co-localisation studies were next carried out in NRVM subjected to sI/R. Figure 5g shows that p53 was co-localised with Brn-3a or Brn-3b in distinct subsets of cells. Interestingly, cells co-expressing Brn-3b and p53 displayed nuclear division and morphological changes that suggest cells may be undergoing mitotic division.

To test if pro-apoptotic genes were co-expressed with Brn-3b following sI/R treatment, co-immunostaining was carried out using *α*-rabbit Noxa or Bax primary antibodies (green) and, in this instance, *α*-goat primary antibody (red) was used to detect Brn-3b. Representative images (Figure 5i) show that following sI/R treatment, nuclear expression of Brn-3b protein was observed in cells that also co-expressed Noxa (i) or Bax (ii), confirming that Brn-3b is co-localised with these apoptotic target genes in response to injury.

### Reducing Brn-3b attenuates the expression apoptotic genes in NRVM following sI/R injury

**Figure 6:**
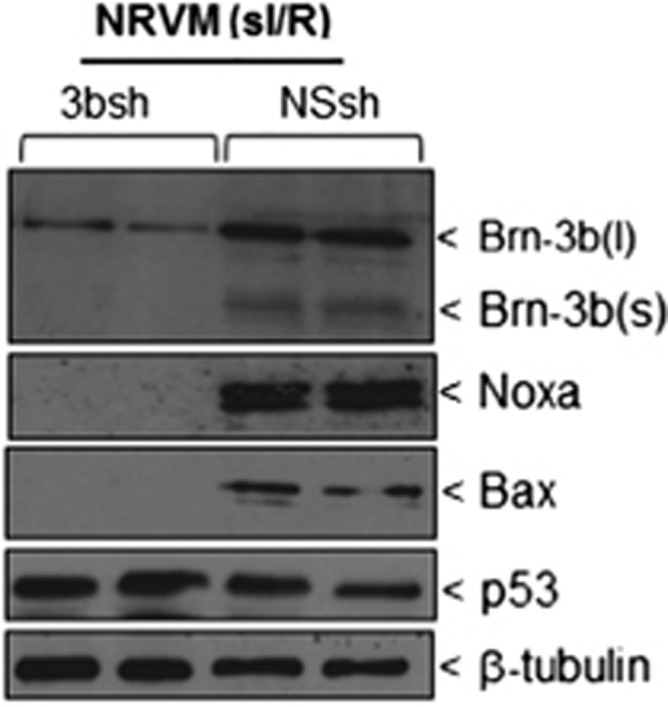
Immunoblot to show changes in Brn-3b, Noxa and Bax proteins in 3b-sh-RNA-infected cells compared with NS controls. p53 protein is also shown whereas *β*-tubulin is used to indicate differences in protein loading between different samples

To test whether Brn-3b was required for maximal expression of pro-apoptotic genes, as previously shown,^24^ lentiviral vectors expressing shRNA to target Brn-3b (3b-sh) or non-silencing control (NS-sh) were used to infect NRVM cultures which were then subjected to sI/R (4 h+6 h). As shown in Figure 6, 3b-shRNA reduced Brn-3b protein levels following sI/R compared with NS-sh cells. Furthermore, 3b-sh also caused the reduction of Bax and Noxa proteins when compared with NS-sh control although p53 expression was relatively unchanged, when compared with *β*-tubulin. These results confirm that Brn-3b was important for maximal expression pro-apoptotic Bax and Noxa proteins in injured cells.

### Brn-3b and p53 expression in H9c2 cells following simulated ischaemia/hypoxia+reoxygenation

**Figure 7:**
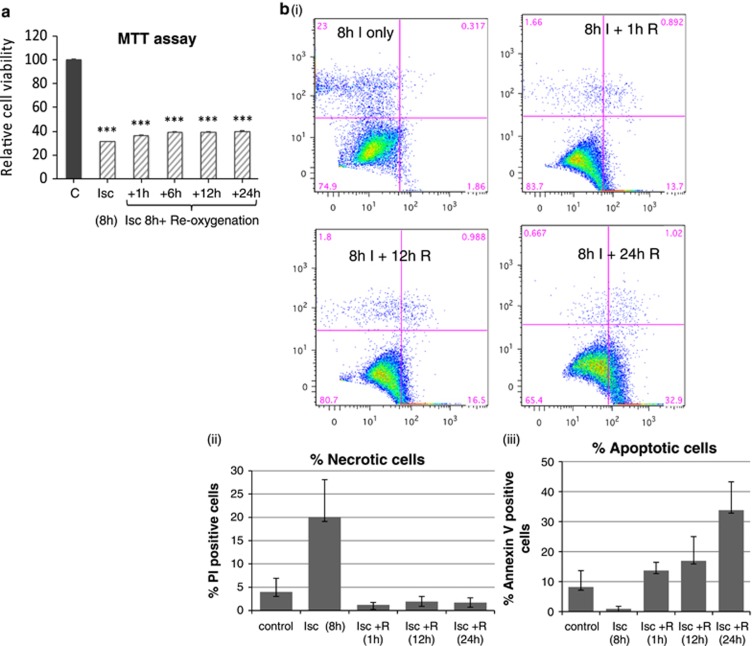

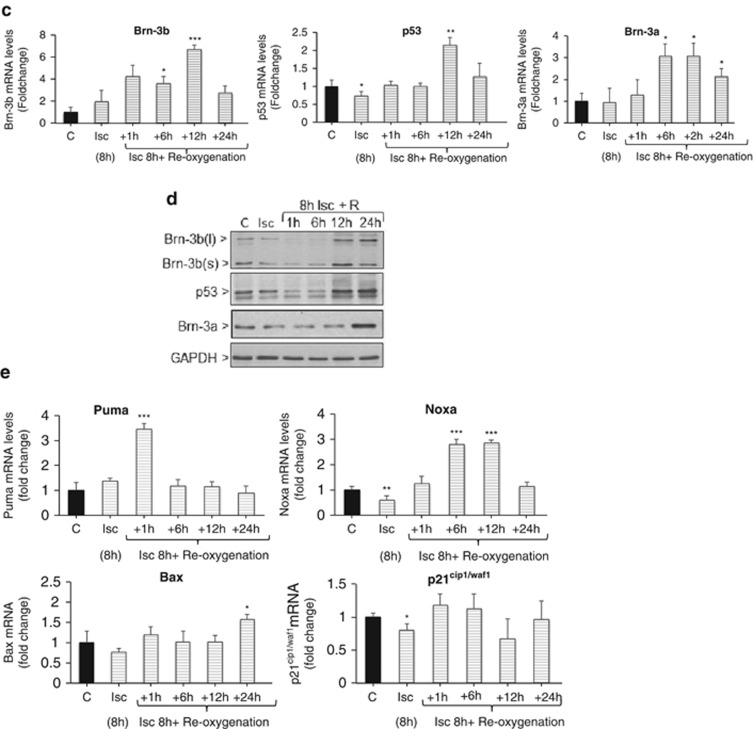
(**a**) Results of MTT assays showing percentage of viable cells in H9c2 cultures subjected to 8 h simulated ischaemia/hypoxia (I) followed by reoxygenation (R) for different times. Values are expressed as percentage decrease compared with untreated control cells (set at 100%) and significant changes are indicated (***P<0.01). (**b**) (i) Representative FACS plots showing the different populations. H9c2 cells that are stained with PI as a marker of necrotic cell death (*y* axis) or annexin V for apoptotic cells (*x* axis) at different times following sI or sI/R. Graphical representation of PI-positive cells undergoing necrosis is shown in (ii), whereas changes in apoptosis, as represented by Annexin V-positive cells are shown in (iii). Significance is indicated by *P<0.05, **P<0.01, ***P<0.001. (**c**) qRT-PCR analysis showing changes in mRNA encoding Brn-3b, p53 or Brn-3a in H9c2 cells treated with ischaemia/hypoxia without or with reoxygenation for different periods. mRNA levels were normalised to GAPDH and differences expressed as fold change relative to the untreated control cells (set at 1). Values represent mean±S.D. from >3 independent experiments at each time point and significant changes are indicated by *P<0.05, **P<0.01, ***P<0.001. (**d**) Representative immunoblots showing changes in Brn-3b, p53 or Brn-3a proteins in untreated and treated H9c2 cells relative to invariant GAPDH protein. (**e**) qRT-PCR analysis of mRNA encoding Puma, Noxa, Bax or p21^cip1/waf1^ in H9c2 cells treated as indicated. mRNA levels were normalised to GAPDH and values expressed as fold change relative to the untreated control cells (set at 1). Mean±S.D. from >3 independent experiments were used to determine statistical significance using Student's *t*-test (*P<0.05, **P<0.01, ***P<0.001)

Similar sI/R studies were carried out in embryonic rat heart-derived H9c2 myoblasts cells and MTT assays showed that sI for 8h caused significant loss of cell viability (~60%) (Figure 7a(i)). Fluorescence activated cell sorting (FACS) analyses demonstrated increased PI staining after sI, (Figure 7b(i)), confirming necrosis as the primary form of cell death under these conditions (Figure 7b(ii)). In contrast, significant increases in Annexin-V staining at 12 h after sI/R (iii) suggest that more cells were undergoing apoptosis in response to reoxygenation injury.

qRT-PCR analysis showed increased Brn-3b and p53 mRNA at 12 h after sI/R in H9C2 cells, whereas Brn-3a mRNA was maximally expressed 24 h after sI/R (Figure 7c). Immunoblots (Figure 7e) showed that proteins were reduced after sI and immediately following sI/R (1–6 h). However, Brn-3b and p53 were increased by 12–24 h after sI/R, whereas Brn-3a was only increased after 24 h (Figure 7d), suggesting some differences in responses between NRVM and H9c2 cells. PUMA protein was increased within 1 h after sI/R, whereas Noxa was maximally expressed by 8–12 h and Bax was increased by 24 h suggesting that these proteins may control cell fate at different times after injury.

### Silencing Brn-3b using SH shRNA reduces pro-apoptotic genes and attenuates apoptosis in H9c2 cells

**Figure 8:**
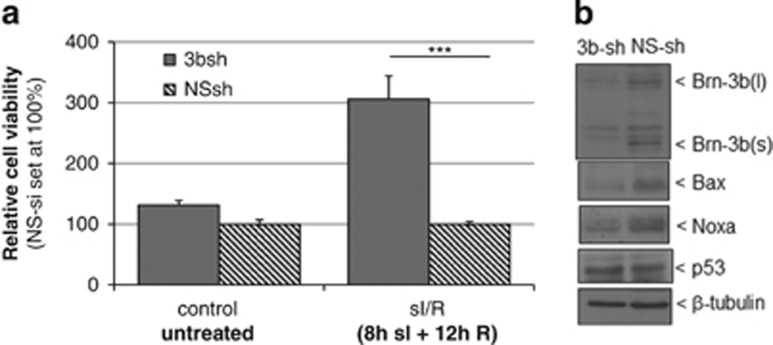
(**a**) Results of MTT assays showing changes in viability of H9c2 cells infected with shRNA to target Brn-3b (3bsh) and subjected to sI/R. Values are expressed as fold change compared with non-silencing shRNA (NSsh), which is set at 1. (**b**) Immunoblot to show changes in Brn-3b and Bax proteins in 3b-sh infected cells compared with NS controls. p53 protein is also shown, whereas b-tubulin is used to indicate differences in protein loading between different samples

We next tested whether targeting Brn-3b affected gene expression and cell viability in H9c2 cells also by using 3b-sh or NS-sh lentiviral vectors. Cells were then subjected to sI/R (8 h+12 h) or left untreated and immunoblots were used to confirm reduced Brn-3b protein in 3b-shRNA-treated cells. MTT assays showed that 3b-shRNA significantly increased cell viability following sI/R when compared with NS-sh control cells but had no effect in control untreated cells (Figure 8a). Loss of Brn-3b following sI/R correlated with reduction of pro-apoptotic Bax and Noxa proteins when compared with NS-sh (Figure 8b) but had no effect on p53 expression. This confirms that Brn-3b was important for maximal expression of these pro-apoptotic proteins in injured cells.

## Discussion

Cardiomyocyte death that occurs following coronary artery occlusion can cause irreversible damage to the heart because these terminally differentiated cells have poor regenerative capacity and are not readily replaced. To attenuate such damage, it is important to understand the complex mechanisms that control cell death in the injured heart. In this study, we have identified Brn-3b and Brn-3a TFs as potential regulators of cardiomyocyte fate following permanent occlusion of the LAD coronary artery, which caused significant loss of viable tissue within 6 h. This model of injury induced apoptotic markers, caspase-3 and -8 in the heart by 6 h, which was sustained at 1 day whereas necroptosis markers, RIP-1/3, were undetectable at 6 h but were induced by 24 h, after injury. This is in line with previous studies in rat hearts in which permanent coronary artery ligation caused apoptotic cell death by 2–4 h after injury but with the peak of necrotic cell death detected at later times (24 h).^9, 46^ Under these conditions, Brn-3b mRNA was increased as early as 1 h after injury, continued to increase at 1 day and although reduced after 1 week, levels remained significantly higher than baseline. On the other hand, Brn-3a mRNA was only increased after 1 day but was reduced by 1 week. Furthermore, analysis of protein expression confirmed these changes but identified distinct expression patterns in relation to the site of injury. Thus, Brn-3a protein was only detected in cardiomyocytes isolated from the non-infarcted regions of the heart but not at the site of injury, and localisation studies showed restricted expression to non-injured regions of the heart. On the other hand, Brn-3b showed more complex, isoform-specific regulation because uninjured myocardium expressed both Brn-3b(l) and Brn-3b(s) isoforms and an additional protein band, slightly larger than Brn-3b(s), that was not expressed in the infarct zone. Although still to be characterised, this may represent some posttranslational modification of the Brn-3b(s) protein expressed in the heart in response to the injury model used. In contrast, only Brn-3b(l) was increased in the injured myocardium, whereas the shorter Brn-3b(s) protein was downregulated. Immunostaining showed Brn-3b expression throughout the injured heart but the antibodies used for these studies recognised both isoforms. As such, the potential distribution of Brn-3b(l) and(s) proteins and implications of their differential expression in the injured heart are still to be determined.

p53 was also induced in this model of cardiac injury but expression was restricted to the infarct zone and the border zone, adjacent to the site of injury. Interestingly, Brn-3a and p53 proteins were mutually exclusive in the injured heart apart from a subset of myocardium adjacent to the infarct zone where these proteins were co-localised. On the other hand, Brn-3b protein, which was detected throughout the injured heart, was co-localised with p53 in myocardium within the infarct zone. Despite conflicting reports regarding its role in cardiomyocyte apoptosis, increasing evidence now supports p53 as a regulator of cell fate in the injured heart because p53 KO mice showed increased survival following coronary artery occlusion, with reduced LV wall thinning and LV rupture compared with wild-type controls.^37^ Similarly, cardiac-specific reduction of p53 using an endogenous p53 antagonist, CHIP, also reduced apoptosis around the infarct border zone after coronary artery ligation and caused less ventricular remodelling during the late phase of MI.^39^ These findings clearly linked p53 expression with increased apoptosis and, interestingly, elevated Brn-3b and p53 in the injured hearts at 1 day after coronary artery ligation correlated with increased apoptotic marker, cleaved caspase-3 and maximal expression of pro-apoptotic proteins, Bax, Noxa and PUMA although anti-apoptotic Bcl-2 and Bcl-X_L_ remained relatively unchanged.

Bax and PUMA proteins were highly expressed in the infarct zone whereas Noxa was detected at the site of injury but also found at lower levels in the non-ischaemic myocardium, suggesting potential contribution to apoptosis at different sites in the heart. These pro-apoptotic proteins have been implicated in cell death following ischaemic/hypoxic injury, with Bax KO mice showing reduced infarct size and less ventricular remodelling after prolonged ischaemia, whereas Langendorf-perfused Bax KO hearts exhibit reduced caspase-3 activity and improved cardiac function after I/R injury.^49, 50^ PUMA is also implicated in cardiomyocyte apoptosis following I/R-induced injury and heart failure and targeted deletion of PUMA in mice has been associated with reduced infarct size because of decreased apoptosis and necrosis.^43, 51^ Although Noxa has not been extensively studied in the heart, its expression is linked to apoptosis in response to hypoxia,^52, 53, 54^ and in this study, we have seen significant increases in Noxa protein following coronary artery ligation in the heart. As Brn-3b co-operates with p53 to enhance apoptotic genes and cell death, it is likely to control myocyte fate in the injured myocardium if co-expressed with p53.

The requirement for Brn-3b in controlling the expression of these pro-apoptotic proteins was supported by studies carried out using *in vitro* cell culture models subjected to sI or sI/R. In line with previous reports, p53 expression was induced in NRVM cultures following sI/R,^7, 36^ whereas Brn-3b was increased in response to sI and continued to increase following sI/R. Importantly, maximal expression of Brn-3b and p53 after 12 h of sI/R correlated with increased Noxa and PUMA expression although with only minimal changes in Bax mRNA. However, relatively high levels of pro-apoptotic genes were observed at baseline in NRVMs, which may reflect stresses sustained by the cells when grown in culture. Despite this, increased Noxa and PUMA may be important for increased apoptosis seen in isolated cardiomyocytes, 12 h after sI/R. Similar studies in H9c2 cells confirmed the relationship between increased Brn-3b and p53 with maximal expression of pro-apoptotic genes, Bax, Noxa and PUMA and confirmed the suitability of this model for analysing the interactions between Brn-3b or Brn-3a and p53, in relation to cells fate determination. Furthermore, targeting Brn-3b using shRNA in NRVM or H9c2 cells was sufficient to reduce Bax and Noxa protein levels, even though p53 remains unchanged. Furthermore, Brn-3b shRNA also attenuated death in H9c2 cells following sI/R, compared with treated cells infected with non-silencing controls. Therefore, Brn-3b is required for maximal expression of pro-apoptotic genes following stresses such as sI/R that stimulate apoptosis. This is in line with previous observations showing that Brn-3b was essential for maximal induction of Bax in neurons because primary cultures from Brn-3b KO mice were highly resistant to apoptotic stimuli which caused significant death in control neurons and expressed reduced Bax mRNA even though p53 expression remain intact.^24^ Thus, Brn-3b may be a crucial regulator of apoptotic cell death in injured cardiomyocytes.

Interestingly, Brn-3b was increased earlier than Brn-3a or p53 *in-vivo* (by 1 h following coronary artery ligation) and *in-vitro* (after sI). This suggests distinct functions for Brn-3b proteins in cardiomyocytes at different times following injury, depending on p53 levels but potential cardiac-specific Brn-3b target genes are still to be identified. Interestingly, this TF can drive cell cycle progression by activating transcription of cell cycle proteins such as cyclin D1 and some cardiomyocytes that co-express Brn-3b and p53 demonstrate morphological characteristics of active mitotic division within the nuclei. Terminally differentiated cells such as cardiomyocytes are thought to respond to injury by abortive attempts to re-enter the cell cycle.^55, 56, 57^ This often precedes cell death referred to as mitotic catastrophe, which is considered as a distinct cell death pathway that results from aberrant control of the cell cycle.^58^ p53 and its target genes, Bax and PUMA, have been implicated in driving mitotic catastrophe.^58, 59^ However, if Brn-3b can affect their expression under these conditions, this may be important for cell fate determination. In this regard, it is possible that early Brn-3b increases in injured cardiomyocytes may stimulate distinct targets, including cell cycle genes, involved in initial responses to injury. However, if the growth-suppressing p53 is induced in Brn-3b-expressing cells, then conflicting signals arise, which cause the cell cycle to be aborted and under these conditions, Brn-3b co-operates with p53 to increase transcription of pro-apoptotic genes, thereby precipitating cell death. Potential roles for Brn-3b in regulating pro-apoptotic genes during mitotic catastrophe may provide a novel method for controlling cell fate. As reducing Brn-3b is sufficient to increase cell viability and reduce pro-apoptotic proteins such as Bax, even though p53 expression is unchanged, these findings confirm that Brn-3b is required for maximal expression of pro-apoptotic target genes in injured myocardium and therefore likely to be very important for mediating such cell fate decisions following MI.

The effects of increased Brn-3a in cardiomyocytes within the non-infarcted regions of the heart are still to be elucidated because Brn-3a target genes, Bcl-2 and Bcl-XL, were not increased in whole hearts but maybe locally increased in surviving cardiomyocytes. However, because Brn-3a can also block p53-mediated apoptosis, it may enhance other p53 target genes, such as NOS3, which is associated with survival in oxygenated myocardium outside the infarct zone.^60^ In this regard, although Brn-3a inhibits p53-mediated apoptic genes, it may cooperate with p53 to regulate distinct target genes that promote survival of specific cell populations that might affect long-term outcome in the injured heart.

Thus, these results provide evidence to support Brn-3b and potentially Brn-3a as key regulators of p53-mediated effects in the heart following injury. The high homology between Brn-3b and Brn-3a in the DNA-binding POU domain (>95%), which also mediates the interaction with p53, suggests that competition between these TFs either for interaction with p53 or binding to promoters of target genes can affect outcome on cell fate. As such, the data showing distinct localisation and co-expression of Brn-3b or Brn-3a with p53 in relation to the injury site is also important for understanding how these factors may control target gene expression and hence the fate of cardiomyocytes, following injury. This is important for elucidating the mechanisms through which these regulators control the fate of cardiomyocytes following injury and will be vital for identifying strategies for increasing cell survival and/or reducing cell death following ischaemic/hypoxic insults that occur in MI.

## Materials and Methods

### Materials

General laboratory reagents were purchased from Merck (Nottingham, UK) and Sigma (Dorset, UK) unless otherwise stated. All tissue culture reagents were supplied by Gibco/Life Technologies (Paisley, UK). Tissue culture plasticware was obtained from Nunc (Paisley, UK) and other general disposable plasticware was from Greiner (Stonehouse, Gloucester, UK) and Corning (supplied by Scientific Laboratory Supplies, Nottingham, UK). Reagents for immunohistochemistry were obtained from Vector Laboratories (Peterborough, UK) and Invitrogen/Life Technologies (Paisley, UK). Primary antibodies were sourced as follows: *α*-Brn-3b (rabbit pAb), *α*-PUMA-(rabbit pAb) and *α*-Noxa (rabbit pAb) (Abcam, Cambridge, UK); *α*-Brn-3b-(goat pAb), GAPDH, (rabbit pAb) and *α*-actin (goat pAb I-19) (Santa Cruz Biotechnology Inc, Dallas, TX, USA); *α*-Brn-3a (rabbit pAb) (Millipore, Dundee, UK), *α*-p53 (sheep pAb) or (mouse mAb) (Calbiochem, Millipore, Billerica, MA, USA), *α*-Bax, (rabbit pAb) and *α*-Cleaved caspase-3, (Rabbit pAb) (Cell Signalling, Danvers, MA, USA), *α*-actinin Mouse (Sigma), *α*-RIP-1 antibody (Cell Signalling 3493), RIP-3 (ab56164), cleaved caspase-8 (CS 4790) and HRP-conjugated secondary Ab from Dako (Cambridgeshire, UK). Lentiviral vector, pGIPZ Brn-3b shRNA (si3B) used to target Brn-3b and control non-silencing (NS) shRNA were obtained from Open Biosystems, Thermo Scientific (Pittsburgh, PA, USA) via UCL Genomics.

### *In vivo* coronary artery ligation

All animal experiments complied with Home Office regulations as detailed in the Guidance on the Operation of the Animals (Scientific Procedures) 1986 and were reviewed and approved by the local Ethics Review Board. Permanent coronary ligation was carried out using 7–14-week old C57BL/6NTac mice as previously described.^61, 62^ Briefly, anaesthetised mice were ventilated by orotracheal intubation prior to performing left thoracotomy to expose the LAD coronary artery which is ligated (using 8-0 nylon suture) in experimental animals or left unligated in sham controls and mice were allowed to recover. Physiological measurements were taken at baseline, after 1 day or 1 week using a Vevo 770 ultrasound imaging system (Fuji film Visual Sonics, Toronto, ON, Canada). At the end of experiments, hearts were excised, rinsed with saline and prepared for subsequent analysis. Western blot analyses were carried out using protein samples obtain from the border zone and the infarct area. For molecular analysis, excised hearts were snap-frozen in liquid nitrogen and stored at −80 °C until use. In some cases, hearts were divided into the infarct from the non-infarct area prior to freezing. Histological assessment of sites of injury in the hearts (e.g., haematoxylin and eosin staining) was carried out using 8-*μ*m sections of hearts that were embedded in OCT compound (Raymond Lamb).

### Triphenyltetrazolium chloride staining

At 6 or 24 h post MI, mice were killed and the heart was quickly excised, sliced into four 1.0-mm-thick sections perpendicular to the long axis of the heart. The sections were then incubated with 1% triphenyltetrazolium chloride at 37 °C for 10 min and then scanned. The infarct area was measured using ImageJ software and myocardial infarct sizes were expressed as a percentage of the left ventricle area.

### Co-immunostaining for protein expression

Protein localisation was analysed on frozen heart sections or NRVM cultured on coverslips. Heart sections were fixed in pre-chilled acetone (5 min) and air-dried at (RT; 5–10 min), followed by permeabilisation in PBS+0.5% Triton-X100 (PBST) for 5 min. For NRVM cells grown on coverslips, treated cells or untreated controls were fixed (4% PFA; 15 mins); washed in 1 × TBS, blocked in PBST+10% goat serum/0.1% w/v BSA for 1 h (RT) followed by incubation with primary antibody (either 4 °C or 1–2 h at RT). After five washes in PBST, Alexa Fluor-conjugated secondary antibodies were incubated for 1 h at RT, followed by 5 × final washes, mounting with DAPI medium (Vector Laboratories). Fluorescence was visualised using Zeiss (Birmingham, UK) Axioscop 2 fluorescent microscope and images captured using Axiovision software (Zeiss).

### Cell models

Primary NRVM cultures were prepared from hearts of 1–2-day-old Sprague-Dawley rats as described.^63^ Briefly, isolated neonatal hearts, minced in ice-cold ADS buffer (116 mM NaCl, 20 mM HEPES, 0.8 mM NaH_2_PO_4_, 5.6 mM glucose, 5.4 mM KCl, and 0.6 mM MgSO_4_) pH 7.35, serially digested in collagenase type 2 (0.5 mg/ml; Worthington Biochemical Corp, Lakewood Township, NJ, USA) and pancreatin (0.6 mg/ml; Sigma), centrifuged, then resuspended in 4 : 1 Dulbecco's Modified Eagle's Medium/Medium 199 (Gibco, Invitrogen Corporation, Auckland, New Zealand) supplemented with 10% horse serum and 5% foetal calf serum +1% penicillin/streptomycin. After 1 h pre-plating (to remove fibroblasts), cells were plated onto gelatine-coated 6-well plates (2 × 10^6^ cells/well) and cultured for 24 h after which medium was replaced with Dulbecco's Modified Eagle's Medium/Medium 199 (4 : 1)+1% foetal calf serum.

H9c2 cells were maintained in Dulbecco's Modified Eagle's Medium with 10% foetal calf serum and 1% penicillin/streptomycin. For experiments, cells were plated onto 6-well plates (5 × 10^5^/well).

### Simulated Ischaemia/Reoxygenation in cell cultures

To simulate ischaemia and reperfusion injury *in vitro*, the culture medium was replaced with ischaemia buffer (137 mM NaCl, 12 mM KCl, 0.49 mM MgCl_2_, 0.9 mM CaCl_2_, 4 mM HEPES, 20 mM sodium lactate, 10 mM 2-Deoxy-glucose, pH 6.2) as previously described^64^ and the cells were incubated in a 37 °C hypoxia chamber with 95% argon, 5% CO_2_ for 4 h (NRVM) or 8 h (H9c2 myoblasts). Following ischaemia/hypoxia, the buffer was replaced with full growth media and the cells were re-oxygenated by transferring into a tissue culture incubator under normal growth conditions (5% CO_2_ at 37 °C), for the indicated times. Control cells were maintained in normal growth media in incubator.

### Gene silencing using ShRNA

To reduce gene expression in H9c2 cells, lentiviral pGIPZ vectors containing short hairpin RNA sequences (shRNA) were used to treat cells for 24 h before treatment with sI/R. The shRNA used were previously shown to effectively reduce Brn-3b (si3B) or Brn-3a (si3a), whereas non-silencing (NS) control shRNA contained a scrambled sequence. shRNA were used at 1.5 × 10^6^ PFU (MOI=0.5) to infect cells prior to treatment.

### RNA extraction, cDNA synthesis and qRT-PCR

Total RNA was isolated from whole hearts, NRVM or H9c2 cells using TRIZOL Reagent (Invitrogen). Snap-frozen whole hearts were homogenised in liquid nitrogen before resuspending in Trizol whereas NRVM and H9c2 cells were harvested in Trizol and processed according to the manufacturer's protocol. DNAse1 treatment was performed using RNAse-free DNAse 1 (Promega, Southampton, UK). cDNA synthesis (20–50 *μ*l reaction) was carried out using RNA Superscript II Reverse Transcriptase (Invitrogen). Real-time qRT-PCR was carried out using 1–2 *μ*l of cDNA. ABI Taqman probes or unique primers (for SYBR Green) were used to quantify each gene and reactions were performed using the Opticon 2 DNA engine thermal cycler (Bio-Rad, Hemel Hempstead, UK). Beta-2 microglobulin or GAPDH housekeeping genes were used to correct for variability between samples. Relative mRNA levels were normalised to GAPDH mRNA and calculated using ΔΔCT method. Mean±S.D. of >5 independent experiments were used for statistical analysis using appropriate packages including Student's *t*-test or Mann-Whitney U-test and significance was shown when *P*<0.05.

### Protein preparation and quantification

Cells were harvested in Laemmli buffer; mouse hearts were pulverised in liquid nitrogen then resuspended in Laemmli buffer. *β*-mercaptoethanol (10% (v/v)) was added to samples prior to boiling (100 °C, 5 min) then centrifuged (13 × *g*, 10 min RT) to remove cellular debris. Total cellular proteins were resolved by polyacrylamide gel electrophoresis (SDS-PAGE) gels (12.5/15%) and immuno blot analysis carried out as previously described.^29^ Briefly, block buffer, PBS-T (phosphate-buffered saline+0.1% Tween-20; 4% milk) was incubated for 1 h; primary Ab (diluted in PBS-T) was incubated for 1 h (RT) or overnight (4 °C) depending on the antibody. After five washes (5 min; RT; PBS-T +0.1% milk), the second Ab was incubated for 1 h and after final washes (4 × 5 min; RT; PBS-T +0.1% milk and 1 × 5 min; PBS-T), signals were developed using enhanced chemiluminescence reagent (Amersham, Bucks, UK). Immunoblots were quantified by analysing bands using densitometry or Image J.

### Cell viability (MTT) assays

Cell viability/proliferation assays were performed using MTT assays, which relies on the ability of live cells to convert the yellow tetrazolium salt (MTT (Sigma) to dark blue, water-insoluble MTT formazan crystals. The conversion in different cultures was detected using colorimetric measurement and the changes in cell viability shown as percentage of control untreated cells. To measure cell death in NRVMs and H9c2 cells after sI/R, monolayer culture of treated cells and untreated controls were rinsed twice with 1 × PBS and 500 *μ*l of MTT solution (0.5 mg/ml in 1 × PBS) was added to each well. After 2 h of incubation at 37 °C, MTT solution was aspirated and 200 *μ*l of DMSO was added to each well. The plates were incubated for 5 min at room temperature with gentle rocking to dissolve the formazan crystals. Fifty microlitres of cell suspension was transferred to the wells of a flat-bottom 96-well plate and the absorbance was measured at 560 nm using a plate reader (TECAN, Reading, UK).

### Flow cytometry

FACS analysis was carried out to assess the fate of H9c2 cells following sI/R because dual-staining protocol with FITC Annexin V and propidium iodide (PI) (BD Pharmingen, Oxford, UK) helped to distinguish cells in early apoptosis (Annexin V positive) or necrosis (PI staining upon loss of membrane integrity). For these studies, cells were seeded as a monolayer in 6-well plates and grown O/N prior to the specified treatment. At the end of experiment, cells were rinsed twice with 1 × HBSS and trypsinised to detached from the wells. Fresh growth medium was added to the cells prior to centrifugation (1000 × *g*; 5 min) and the pellet was resuspended in 100–300 *μ*l of 1 × binding buffer. One hundred microlitres of the cell suspension was transferred to fresh 1.5 ml eppendorf tubes and 5 *μ*l of FITC-conjugated Annexin V and/or 5 *μ*l PI were added to cells and incubated for 30 min at room temperature, mixing gently every 10 min. One millilitre of FACS buffer (0.5% w/v BSA and 0.1% sodium azide in 1 × PBS) was then added to each tube and the cell suspensions were centrifuged at 1000 × *g* for 5 min. To remove residual Annexin V and PI in the tubes, a washing step was carried out by dissolving the pellets in 1 ml of FACS buffer, followed by centrifugation at 1000 × *g* for 5 min. The pellet was then resuspended in 350 *μ*l of FACS buffer and the entire cell suspension was transferred to FACS tubes. Analysis was performed using the FACSCalibur flow cytometer (Becton Dickinson, Oxford, UK). Data were analysed using the FlowJo data analysis software package (TreeStar, Ashland, OR, USA).

## Abbreviations

MI: myocardial infarction
RIP-1/3: receptor-interacting proteins, RIP1 and RIP3
TFs: transcription factors
POU: Pit-Oct-Unc
Brn-3b: POU4F2/Brn-3b
Brn-3a: POU4F1/Brn-3a
Brn-3b(l): longer Brn-3b protein isoform
Brn-3b(s): short Brn-3b protein
I/R: ischaemia/reperfusion injury
sI: simulated ischaemia/hypoxia
sI/R: simulated ischaemia/hypoxia+reoxygenation
LAD: left anterior descending coronary artery
NRVM: neonatal rat ventricular cardiomyocytes
MTT: Methylthiazolyldiphenyl-tetrazolium bromide
FACS: Fluorescence Activated Cell Sorting
CHIP: carboxyl terminus of Hsp70-interacting protein
